# PD-L1 Antibody Pharmacokinetics and Tumor Targeting in Mouse Models for Infectious Diseases

**DOI:** 10.3389/fimmu.2022.837370

**Published:** 2022-03-10

**Authors:** Gerwin G. W. Sandker, Gosse Adema, Janneke Molkenboer-Kuenen, Peter Wierstra, Johan Bussink, Sandra Heskamp, Erik H. J. G. Aarntzen

**Affiliations:** ^1^ Department of Medical Imaging, Radboud Institute for Molecular Life Sciences, Radboud University Medical Center, Nijmegen, Netherlands; ^2^ Department of Radiation Oncology, Radboud Institute for Molecular Life Sciences, Radboud University Medical Center, Nijmegen, Netherlands

**Keywords:** PD-L1, *Staphylococcus aureus*, *Candida albicans*, lipopolysaccharide, nuclear imaging (SPECT), antibody, cancer, infectious diseases

## Abstract

**Background:**

Programmed death-ligand 1 (PD-L1) regulates immune homeostasis by promoting T-cell exhaustion. It is involved in chronic infections and tumor progression. Nuclear imaging using radiolabeled anti-PD-L1 antibodies can monitor PD-L1 tissue expression and antibody distribution. However, physiological PD-L1 can cause rapid antibody clearance from blood at imaging doses. Therefore, we hypothesized that inflammatory responses, which can induce PD-L1 expression, affect anti-PD-L1 antibody distribution. Here, we investigated the effects of three different infectious stimuli on the pharmacokinetics and tumor targeting of radiolabeled anti-PD-L1 antibodies in tumor-bearing mice.

**Materials/Methods:**

Anti-mouse-PD-L1 and isotype control antibodies were labelled with indium-111 ([^111^In]In-DTPA-anti-mPD-L1 and [^111^In]In-DTPA-IgG2a, respectively). We evaluated the effect of inflammatory responses on the pharmacokinetics of [^111^In]In-DTPA-anti-mPD-L1 in RenCa tumor-bearing BALB/c mice in three conditions: lipopolysaccharide (LPS), local *Staphylococcus aureus*, and heat-killed *Candida albicans*. After intravenous injection of 30 or 100 µg of [^111^In]In-DTPA-anti-mPD-L1 or [^111^In]In-DTPA-IgG2a, blood samples were collected 1, 4, and 24 h p.i. followed by microSPECT/CT and *ex vivo* biodistribution analyses. PD-L1 expression, neutrophil, and macrophage infiltration in relevant tissues were evaluated immunohistochemically.

**Results:**

In 30 µg of [^111^In]In-DTPA-anti-mPD-L1 injected tumor-bearing mice the LPS-challenge significantly increased lymphoid organ uptake compared with vehicle controls (spleen: 49.9 ± 4.4%ID/g versus 21.2 ± 6.9%ID/g, p < 0.001), resulting in lower blood levels (3.6 ± 1.6%ID/g versus 11.5 ± 7.2%ID/g; p < 0.01) and reduced tumor targeting (8.1 ± 4.5%ID/g versus 25.2 ± 5.2%ID/g, p < 0.001). Local *S. aureus* infections showed high PD-L1^+^ neutrophil influx resulting in significantly increased [^111^In]In-DTPA-anti-mPD-L1 uptake in affected muscles (8.6 ± 2.6%ID/g versus 1.7 ± 0.8%ID/g, p < 0.001). Heat-killed *Candida albicans* (Hk-*C. albicans*) challenge did not affect pharmacokinetics. Increasing [^111^In]In-DTPA-anti-mPD-L1 dose to 100 µg normalized blood clearance and tumor uptake in LPS-challenged mice, although lymphoid organ uptake remained higher. Infectious stimuli did not affect [^111^In]In-DTPA-IgG2a pharmacokinetics.

**Conclusions:**

This study shows that anti-PD-L1 antibody pharmacokinetics and tumor targeting can be significantly altered by severe inflammatory responses, which can be compensated for by increasing the tracer dose. This has implications for developing clinical PD-L1 imaging protocols in onco-immunology. We further demonstrate that radiolabeled anti-PD-L1 antibodies can be used to evaluate PD-L1 expression changes in a range of infectious diseases. This supports the exploration of using these techniques to assess hosts’ responses to infectious stimuli.

## Introduction

Immune checkpoint molecules play a vital role in immune homeostasis; they prevent autoimmune disease and maintain tissue integrity during inflammatory responses. Programmed death-ligand 1 (PD-L1, also CD274) is a key immune checkpoint, with a dynamic expression profile on a broad range of tissues (1). It promotes effector T-cell exhaustion when ligating its receptor programmed death-1 (PD-1), thereby down-tuning the adaptive immune response. In cancer patients, PD-L1 expressed by tumor or activated immune cells promotes immune escape (2, 3). Blocking PD-L1 with therapeutic antibodies results in increased anti-tumor immunity in various cancer types and has revolutionized cancer immunotherapy (4). In response to infection, increased levels of local or systemic proinflammatory cytokines, e.g., IFN-γ and TNF-α, induce the upregulation of PD-1/PD-L1 expression (5–7). Paralleling onco-immunology, T-cell dysfunction and exhaustion mediated by increased PD-1/PD-L1 expression in patients with unresolved chronic infections or sepsis result in ineffective microbial clearing and increased mortality (5, 6, 8). In preclinical sepsis models, PD-1 and PD-L1 blocking monoclonal antibodies (mAb) have been shown to reinvigorate the immune system, thereby enhancing bacterial clearance and improving survival (9, 10). These results prompted clinical studies evaluating the efficacy of PD-1 or PD-L1 targeting mAbs in patients with sepsis (11, 12).

Nuclear imaging using radiolabeled anti-PD-L1 antibodies allows for non-invasive, sensitive, and quantitative assessments of PD-L1 expression on a whole-body scale (13–16). Various preclinical studies in onco-immunology have demonstrated the feasibility of this approach in immunocompetent mouse models (17–19). Moreover, recent clinical studies using a radiolabeled anti-PD-L1 antibody (14, 20), adnectin (15), peptide (21), or nanobody (22) demonstrated that nuclear imaging using PD-L1 targeting tracers can assess PD-L1 expression *in vivo* (18). Accumulation of the radiolabeled anti-PD-L1 antibodies was also observed in tissues with physiological PD-L1 expression (e.g., spleen, lymph nodes, and bone marrow) as well as in inflammatory sites (14). In general, the spleen is the main organ for the accumulation of radiolabeled antibodies (23). During systemic responses to infection, PD-L1 expression in the spleen is upregulated (10). Therefore, we hypothesized that inflammatory responses that induce local or systemic PD-L1 expression can affect anti-PD-L1 antibody biodistributions. Furthermore, no study has performed an in-depth investigation of PD-L1 expression or imaging in infectious disease models.

The aim of our study was to assess changes in PD-L1 expression in response to a range of inflammatory stimuli and how these would influence imaging of PD-L1 expression in the tumor. Severe-to-mild bacterial infection was mimicked by inducing systemic inflammatory responses with lipopolysaccharide (LPS), or local responses with *Staphylococcus aureus* infection or heat-killed *Candida albicans* (Hk-*C. albicans*) in tumor-bearing mice. Analysis of blood clearance and *in vivo* biodistribution of different doses of indium-111-labeled anti-mPD-L1 antibodies showed that inflammatory responses can significantly alter the physiological PD-L1 expression in lymphoid organs, both locally and systemically. This resulted in accelerated blood clearance and decreased tumor targeting of radiolabeled PD-L1 antibodies.

## Materials and Methods

### Study Design

The effect of three clinically relevant infection models on the pharmacokinetics of anti-mPD-L1 antibodies was investigated. For this purpose, RenCa tumor-bearing female BALB/c mice were injected with 1) LPS intraperitoneal, 2) *S. aureus* intramuscular, 3) Hk-*C. albicans* intraperitoneal, 4) vehicle intramuscular, or 5) vehicle intraperitoneal. Subsequently, mice were injected intravenously with 30 µg of [^111^In]In-DTPA-anti-mPD-L1 mAb (^111^In-mPD-L1, previously determined as the optimal tracer dose for PD-L1 imaging) (24). Next, the effect of antibody dosing on the pharmacokinetics in these infection models was examined by injecting identical groups with 100 µg of ^111^In-mPD-L1. Finally, to assess whether uptake was PD-L1 specific, subgroups of mice were injected with 30 or 100 µg of ^111^In-labeled non-biologically affine IgG2a. For all conditions, the antibody blood concentrations were assessed by collecting blood samples at several time points following tracer injection. Furthermore, the distribution of the radiolabeled antibodies was assessed by single-photon emission CT (SPECT)/CT and *ex vivo* biodistribution analysis. PD-L1 tissue expression was investigated immunohistochemically. All *in vivo* experiments were approved by the Animal Welfare Body of the Radboud University, Nijmegen, and the Central Authority for Scientific Procedures on Animals and were conducted in accordance with the principles laid out by the Dutch Act on Animal Experiments (2014).

### Cell Culture

RenCa murine renal cell carcinoma cells (CLS, 400321) were cultured in Roswell Park Memorial Institute (RPMI) 1640 (Gibco, Life Technologies Limited, Paisley, UK), supplemented with 10% fetal calf serum (FCS; Sigma-Aldrich Chemie BV, Zwijndrecht, the Netherlands) and 2 mmol/L glutamine (Gibco, Grand Island, NY, USA) at 37°C in a humidified atmosphere containing 5% CO_2_. Cells were tested and found negative for mycoplasma and mouse pathogens. The maximum number of passages between thawing and tumor cell inoculation was five.

### Tumor-Bearing Mice

Female BALB/c mice (n = 126, 10–12 weeks, Janvier Labs, Le Genest-Saint-Isle, France) were housed under specific-pathogen-free conditions in individually ventilated cages with a filter top (Blue line IVC, Techniplast, Buguggiate, VA, Italy) in which cage enrichment was present and food and water were available *ad libitum*. Mice (n = 121) were injected subcutaneously (right flank) with 0.5 × 10^6^ RenCa cells in 200 µl of RPMI 1640. Tumor size was measured using a caliper twice weekly. When mean tumor volume reached approximately 0.2 cm^3^, mice were block-randomized into the following groups; 1) LPS intraperitoneal, 2) *S. aureus* intramuscular, 3) Hk-*C. albicans* intraperitoneal, 4) vehicle control intraperitoneal, or 5) vehicle control intramuscular. A total of 21 mice were excluded from analysis for the following reasons: no discernable tumor present at the time of dissection (n = 16), a humane endpoint was reached (n = 3), the animal deceased before the end of the experiment (n = 1), or presence of a large concavity in the tumor (n = 1).

### Preparation and Administration of Infection Model Agents

#### Lipopolysaccharide

LPS (L6529, Sigma-Aldrich, St. Louis, MO, USA) was dissolved in physiological saline (0.9% NaCl, Braun Melsungen AG, Melsungen, Germany) and stored at –20°C at a concentration of 1 mg/ml. Solutions were thawed, and physiological saline was added to a final concentration of 0.075 mg/ml. LPS was injected intraperitoneally (0.6 mg/kg body weight) 24 h before radiolabeled antibody injection, the previously determined optimal time point (24).

#### Staphylococcus aureus

*S. aureus* (substrain 25923, ATCC, Manassas, VA, USA) were seeded in Columbia III agar containing 5% sheep blood (BD Biosciences, San Jose, CA, USA) and incubated overnight at 37°C. Next, in duplicate, 3 to 5 colonies *S. aureus* were transferred to 4.5 ml of brain heart infusion (BHI) broth (BD) in 15-ml falcon tubes and incubated overnight at 37°C. The following day, the suspension was spun down at 1,000*g*, and the pellet was resuspended in 5 ml of NaCl 0.9% (Laboratoire Aguettant, Lyon, France); subsequently, the content of both tubes were pooled (10 ml, 1 × 10^9^ colony-forming units (CFU)/ml). This suspension was transported at –80°C on dry ice and thawed 1 h later right before use. The bacterial suspension was mixed 1:1 v/v with heparinized homologous blood collected by heart puncture from isoflurane-anesthetized donor mice. The exact CFU count was calculated from a titration series of the sample, which was incubated overnight on agar at 37°C. Mice were anesthetized using isoflurane, and *S. aureus* (50 µl, with CFUs ranging from 0.8 × 10^7^ to 4.5 × 10^7^) was injected in the left calf muscle 48 h before radiolabeled antibody injection for the infection and subsequent inflammatory response to develop sufficiently (25).

#### Heat-Killed *Candida albicans*

*C. albicans* (substrain UC 820, ATCC) were incubated overnight in Sabouraud broth at 30°C and subsequently killed by heating at 56°C for 30 min, and the preparation was kindly provided by M. Jaeger and M. Netea, Radboudumc Nijmegen (26). The suspension was diluted with sterile pyrogen-free phosphate-buffered saline (PBS) to a final concentration of 5 × 10^7^ CFU/ml. Hk-*C. albicans* was injected intraperitoneally (0.5 × 10^7^ CFU Hk-*C. albicans* in 100 μl of PBS) 24 h before radiolabeled antibody injection for antifungal immune responses to develop (27).

#### Vehicle Controls

For the groups receiving intraperitoneal injection, physiological saline (0.9% NaCl) was used as a vehicle control (for the LPS and Hk-*C. albicans* model groups) and injected 24 h before radiolabeled antibody injection. For the intramuscular *S. aureus* group, vehicle control was a mixture of autologous blood:physiological saline 1:1 v/v (prepared according to the protocol for the *S. aureus* infection). The mixture was injected 48 h before radiolabeled antibody injection.

### Radiolabeling and Antibody Injection

Rat IgG2b anti-murine PD-L1 (mPD-L1, clone 10F.9G2, Bio X Cell, Lebanon, NH, USA) and rat IgG2a non-biological affine control (IgG2a, clone 2A3, Bio X Cell) antibodies were conjugated with isothiocyanatobenzyl-diethylenetriaminepentaacetic acid (p-SCN-Bn-DTPA, Macrocyclics, Plano, TX, USA), as described previously (13, 24). DTPA-conjugated antibodies were labeled with indium-111 chloride (^111^InCl_3_, Curium, Helsinki, Finland) as follows: DTPA-anti-mPD-L1 or DTPA-IgG2a were incubated with ^111^InCl_3_ in a ratio of 1 MBq per µg antibody in 0.5 M of MES buffer (pH 5.4) at room temperature (RT) for 20 min. Thereafter, unincorporated ^111^In was chelated by adding 1/10th of the labeling volume of 50 mM of ethylenediaminetetraacetic (EDTA) in PBS to reach a final concentration of 5 mM of EDTA. The labeling efficiencies were evaluated using instant thin-layer chromatography (iTLC), using silica gel impregnated glass microfiber membrane chromatography strips (SGI0001, Agilent Technologies, Santa Clara, CA, USA) and 0.1 M, pH 6.0, of citrate buffer (Sigma-Aldrich) as a mobile phase. The activity profiles were captured with photosensitive plates (Fuji MS, Cytiva, Marlborough, MA, USA) and analyzed using a phosphor imager (Typhoon FLA 7000, GE, Chicago, IL, USA). When labeling efficiency was ≥92.5%, pH was adjusted by adding 1/10th volume 10× PBS (pH 7.4). When labeling efficiency was below 92.5%, non-labeled ^111^In was removed using PD10 buffer exchange columns (Cytiva) and PBS as eluant following the manufacturer’s protocol. Moreover, to lower the salt concentration of all IgG2a labeling reactions and subsequent injection fluids, buffers were exchanged to PBS using PD10 columns directly after the radiolabeling procedure. Subsequently, the desired concentration of either 30 or 100 µg of antibody per 200 µl was reached by adding the appropriate amount of unlabeled antibody and volume of sterile pyrogen-free PBS. The radiochemical purity of the injected products exceeded 92.5% for all experiments. For each infection model groups, mice were injected in the lateral caudal vein with 200 µl of PBS containing one of the following; a) 30 µg of [^111^In]In-DTPA-anti-mPD-L1 mAb (^111^In-mPD-L1, specific activity (A_s_): 0.34–0.57 MBq/µg), b) 100 µg of ^111^In-mPD-L1 (A_s_: 0.102–0.172 MBq/µg), c) 30 µg of [^111^In]In-DTPA-IgG2a (^111^In-IgG2a, A_s_: 0.0063 MBq/µg), or d) 100 µg of ^111^In-IgG2a (A_s_: 0.0055 MBq/µg). Heat lamps warmed the mice prior to i.v. injections. The *in vitro* and *in vivo* characterization of ^111^In-mPD-L1 and ^111^In-rIgG2a has been reported previously (13, 24). During all experiments, researchers and biotechnicians were blinded for group allocation.

### Pharmacokinetics, *Ex Vivo* Biodistribution, and MicroSPECT/CT Imaging

To assess the blood clearance of ^111^In-mPD-L1 over time, blood samples of all mice were collected using 25-µl capillary tubes (Hirschmann, Eberstadt, Germany) following lateral caudal venipuncture, at 1, 4, and 24 h post tracer injection. When lateral caudal venipuncture proved unsuccessful, blood samples were obtained by saphenous venipuncture. At 24 h post tracer injection, all mice were euthanized by CO_2_/O_2_ asphyxiation. In each group, 2 mice were used for SPECT/CT imaging to visualize the *in vivo* biodistribution of ^111^In-mPD-L1 with SPECT/CT (U-SPECT II/CT, MILabs, Houten, Netherlands) using the following acquisition settings: 25-min acquisition time, 1.0-mm-diameter pinhole mouse high sensitivity collimator, and CT parameters of 160-µm spatial resolution, 615 µA, and 65 kV. Data were reconstructed using the MILabs software (version 2.04) using the following settings: energy windows at 171 keV (range 154 to 188 keV) and 245 keV (range 220 to 270 keV), 1 iteration, 16 subsets, and a 0.4-mm voxel size. The Inveon Research Workplace software package (version 4.1) was used to create maximum intensity projections (MIPs). For all mice, directly following euthanasia or SPECT/CT scanning, tumor and other tissues of interest (spleen, left and right inguinal lymph nodes, brown adipose tissue, bone marrow, thymus, duodenum, liver, kidney, heart, lung, pancreas, stomach, colon, bone, muscle, and when available *S. aureus*-infected or vehicle control-injected muscle) were collected for *ex vivo* biodistribution analysis. Blood and tissue samples were weighed (XPE105DR, Mettler Toledo, Columbus, OH, USA) and measured with a well-type gamma-counter (Wallac 2480 Wizard, Perkin Elmer, Waltham, MA, USA). For reference, aliquots of injection fluid were measured concomitantly. Antibody accumulation was calculated as a fraction of the injected dose and normalized for tissue weight [percentage injected dose per gram tissue (%ID/g)]. Tumor, the spleen, and *S. aureus*-infected and vehicle-injected muscle were stored in 4% formalin in PBS for subsequent immunohistochemical evaluation.

### Immunohistochemistry

Formalin-fixed paraffin-embedded tissue sections (5 µm) of tumor and the spleen, and *S. aureus*-infected and vehicle control muscle were evaluated by immunohistochemistry for PD-L1 expression and PD-L1, F4/80, and Ly6G expression respectively. All sections were deparaffinized and rehydrated. For F4/80, sections were post fixated with 4% formalin in PBS for 10 min at RT. Antigen retrieval (AR) was performed in either 10 mM of sodium citrate buffer (pH 6.0) at 96°C for 10 min for both PD-L1 and Ly6G, or at 37°C for 2 h followed by 0.075% trypsin in PBS for 7 min at 37°C for F4/80. Endogenous peroxidases were blocked before AR with 3% H_2_O_2_ (107209, Merck, Darmstadt, Germany) in methanol for 20 min for F4/80, or after AR with 3% H_2_O_2_ in PBS for 10 min for both PD-L1 and Ly6G. For PD-L1, endogenous biotin was blocked using a biotin/avidin blocking set (SP-2001, Vector, Burlingame, CA, USA). Next, for PD-L1 and F4/80, blocking was performed with 10% normal rabbit serum (NrabS, BDC-10112, Bodinco, Alkmaar, Netherlands) and for Ly6G with 20% normal goat serum (5095, Bodinco) in PBS for 30 min. Primary antibody incubation was performed with either goat-a-murine PD-L1 (AF1019, R&D Systems, Minneapolis, MN, USA) diluted 1:500 in PBS/1% bovine serum albumin (BSA; Sigma, Darmstadt, Germany) overnight at 4°C, rat-anti-F4/80 (BM8, Invitrogen, Carlsbad, CA, USA) diluted 1:1,000 in PBS/2% NrabS and incubated overnight at 4°C, or rat-anti-mouse-Ly6G (BP0075-1, Bio X Cell) diluted 1:1,000 in PBS+1% BSA and incubated for 2 h at RT. Thereafter, tissues were incubated for 30 min at RT with the following secondary antibodies: biotinylated-rabbit-anti-goat (E0466, DAKO, Glostrup, Denmark) diluted 1:400 in PBS/1% BSA, goat-a-Rat-peroxidase (A9037, Sigma) diluted 1:100 in PBS/1% BSA, or biotinylated rabbit-anti-rat-IgG (BA-4001, Vector) 1:200 dilution in PBS-2% NrabS, for the PD-L1, Ly6G, or F4/80, respectively. Next, for PD-L1 and F4/80, horseradish peroxidase-Biotin/Streptavidin Complex (PK-6100, Vectastain) diluted 1:250 in PBS/1% BSA for 30 min at RT was used. Finally, all tissues were incubated with bright DAB (B-500, Immunologic, Duiven, the Netherlands) for 8 min, followed by nuclei blueing with hematoxylin (4085-9001, Klinipath/VWR, Olen, Belgium), and dehydrated with ethanol (4099-9005, Klinipath/VWR) and xylene (4055-9005, Klinipath/VWR). Sections were mounted with a coverslip using Permount (SP15-500, Fisher, Hampton, NH, USA). Consecutive sections to the PD-L1-, F4/80-, and Ly6G-stained sections were stained for morphological reference with H&E Y (1.15935.0100, Merck).

### Statistical Analysis

Differences in tissue uptake and blood concentrations of both doses ^111^In-mPD-L1 and ^111^In-IgG2a were tested for significance using two-way ANOVAs with a Bonferroni *post-hoc* test. Values are presented as mean ± SD. Statistical significance was defined as a p-value below 0.05. Statistical analyses were performed using GraphPad Prism version 5.03 for Windows.

## Results

### Lipopolysaccharide Significantly Changes ^111^In-mPD-L1 Pharmacokinetics and Tumor Targeting

**Figure 1:**
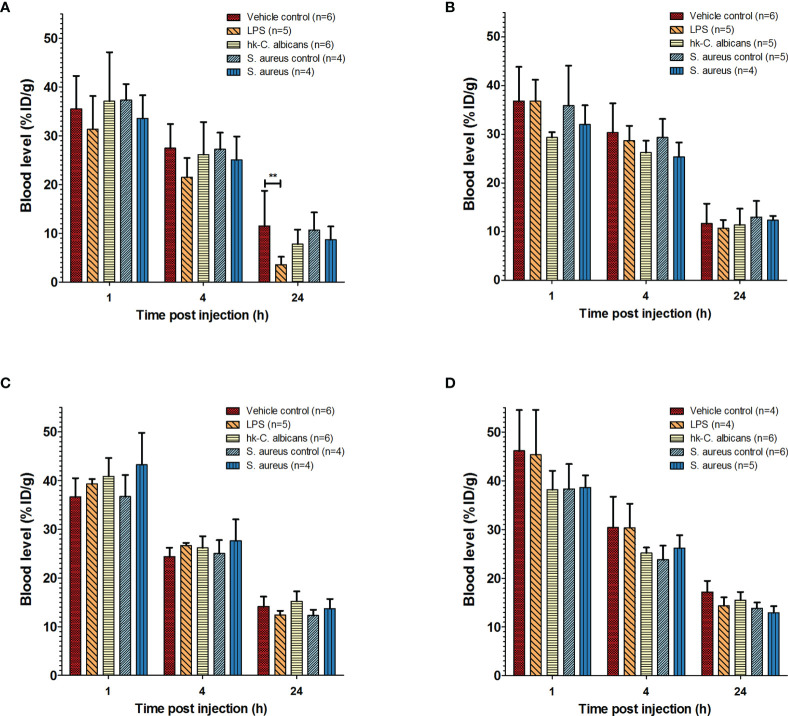
Pharmacokinetics of ^111^In-mPD-L1 and ^111^In-IgG2a in a range of infection models. Pharmacokinetics of 30 µg of ^111^In-mPD-L1 **(A)**, 100 µg of ^111^In-mPD-L1 **(B)**, 30 µg of ^111^In-IgG2a **(C)**, and 100 µg of ^111^In-IgG2a **(D)** in RenCa tumor-bearing BALB/c mice immune stimulated with the following conditions: vehicle control, LPS, Hk-*C. albicans*, *Staphylococcus aureus* i.m. infected, and vehicle i.m. control. ^111^In-mPD-L1 and ^111^In-IgG2a blood concentrations 1, 4, and 24 h post antibody injection are given in % injected dose per gram of blood (%ID/g). Differences in blood levels were tested for significance using two-way ANOVAs with a Bonferroni *post-hoc* test (**p < 0.01) (see legend in figure for number of animals per group). LPS, lipopolysaccharide; Hk-*C. albicans*, heat-killed *Candida albicans*.

**Figure 2:**
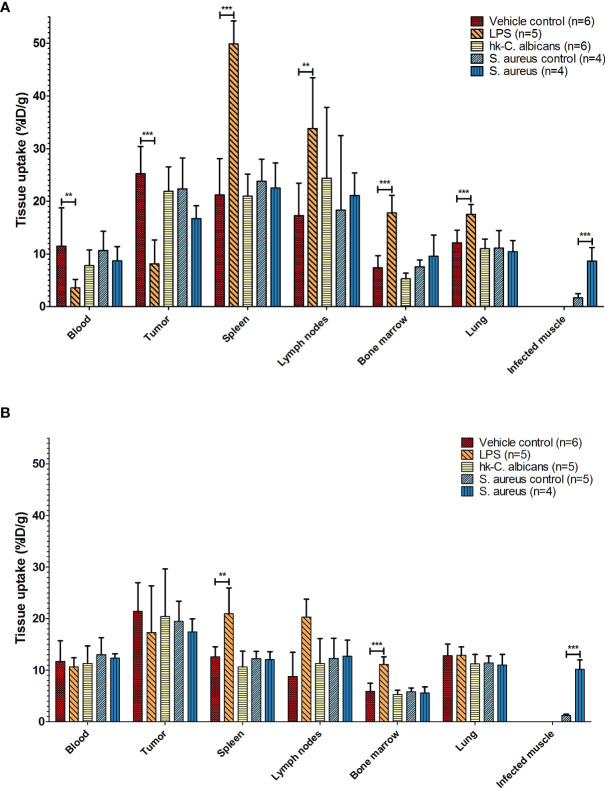
Biodistribution of ^111^In-mPD-L1 in different infection models. *Ex vivo* biodistribution of 30 µg and 100 µg of ^111^In-mPD-L1 antibody doses in RenCa tumor-bearing BALB/c mice injected with vehicle control or immune stimulated with LPS, Hk-*C. albicans*, or *Staphylococcus aureus*, at 24 h post injection. Data for tissues not shown can be found in **Supplementary Table 1**. **(A)** In 30 µg of ^111^In-mPD-L1-injected mice, immune stimulation with LPS significantly affects ^111^In-mPD-L1 blood clearance, lymphoid, and tumor uptake. In *S. aureus*-infected muscle, significantly increased uptake of ^111^In-mPD-L1 was observed. **(B)** For 100 µg of ^111^In-mPD-L1-injected mice, LPS stimulation increased uptake in lymphoid organs, but no effect was observed on blood levels or tumor targeting. Significant uptake of ^111^In-mPD-L1 can be observed in *S. aureus*-infected muscles. Biodistribution of neither ^111^In-mPD-L1 dose was affected by Hk-*C. albicans* stimulation. ^111^In-mPD-L1 tissue uptake is given in % injected dose per gram of tissue (%ID/g), and differences in uptake were tested for significance using two-way ANOVAs with a Bonferroni *post-hoc* test (**p < 0.01, and ***p < 0.001) (see legend in figure for number of animals per group). LPS, lipopolysaccharide; Hk-*C. albicans*, heat-killed *Candida albicans*.

The effect of LPS on the pharmacokinetics of ^111^In-mPD-L1 in tumor-bearing mice is presented in **Figures 1** and **2**, and the biodistribution data of all collected organs can be found in **Supplementary Table 1**. Increased PD-L1 antibody clearance from blood was observed in LPS-challenged mice injected with 30 µg of ^111^In-mPD-L1, compared with control mice (24 h p.i.: 3.6 ± 1.6%ID/g versus 11.5 ± 7.2%ID/g, respectively, p < 0.01; see also **Supplementary Table 1**). Furthermore, accumulation of ^111^In-mPD-L1 in the spleen was more than twofold higher in LPS-challenged compared with control mice (49.9 ± 4.4%ID/g versus 21.2 ± 6.9%ID/g, respectively, p < 0.001). Increased PD-L1 antibody uptake was also observed in lymph nodes (33.8 ± 9.6%ID/g versus 17.3 ± 6.1%ID/g, p < 0.01), bone marrow (17.8 ± 3.3%ID/g versus 7.4 ± 2.3%ID/g, p < 0.001), and, to a lesser extent, other tissues with physiological PD-L1 expression, such as the lungs, heart, duodenum, and brown adipose tissue. The increased uptake of ^111^In-mPD-L1 in primary and secondary lymphoid organs occurred at the expense of tumor uptake, which was only 8.1 ± 4.5%ID/g in LPS-challenged mice versus 25.2 ± 5.2%ID/g in control mice (p < 0.001).

**Figure 3:**
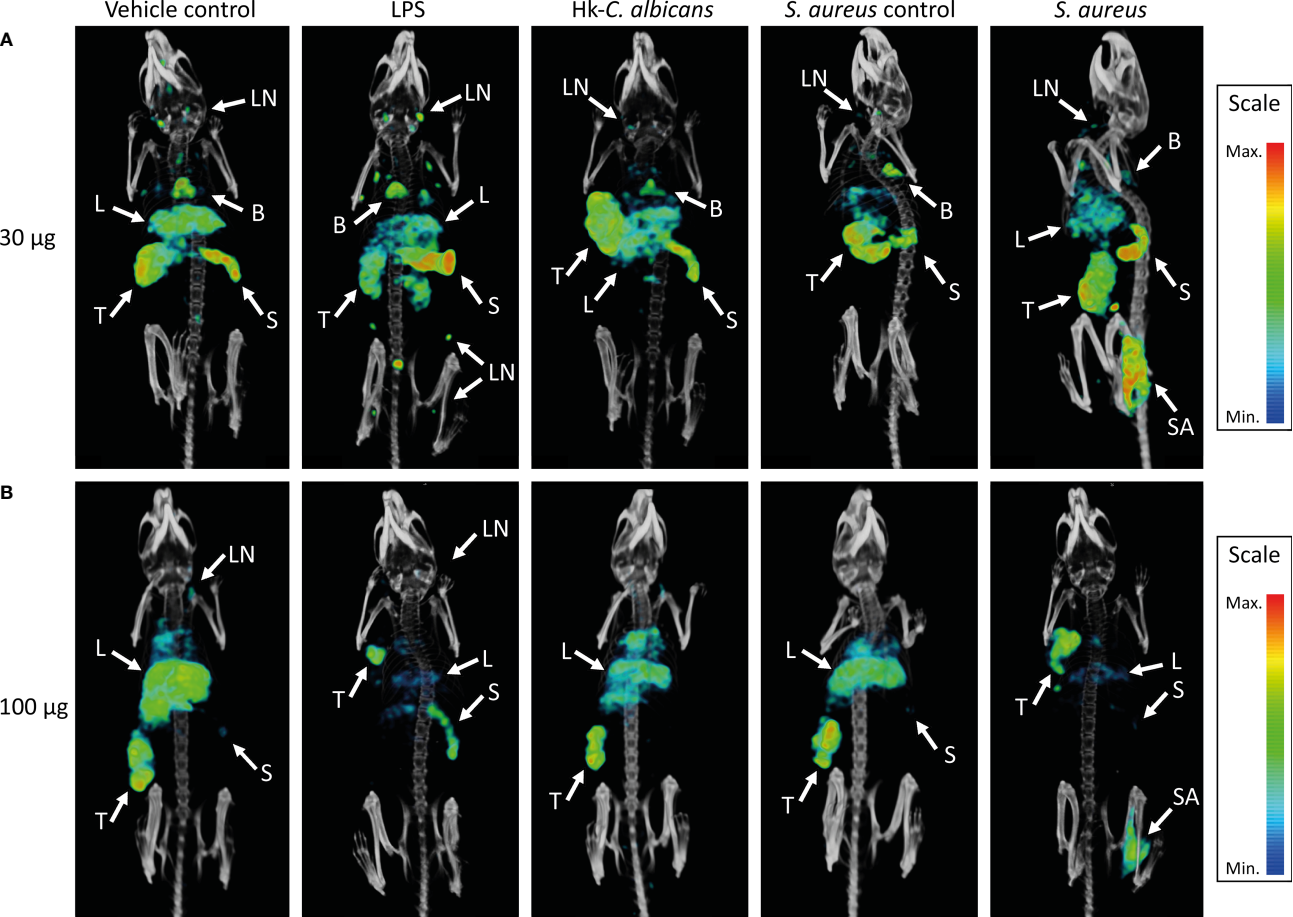
SPECT/CT images showing the *in vivo* distribution of ^111^In-mPD-L1 in vehicle-injected and LPS-stimulated, *Staphylococcus aureus*-infected, or Hk-*C. albicans*-stimulated RenCa tumor-bearing mice. Representative MIPs of vehicle control, LPS-stimulated, *S. aureus*-infected, and Hk-*C. albicans-*stimulated RenCa tumor-bearing BALB/c mice, 1 day after injection 30 µg of ^111^In-mPD-L1 **(A)** or 100 µg of ^111^In-mPD-L1 **(B)**. Tissue uptake of ^111^In-mPD-L1 is visualized with MIP thresholded microSPECT/CT images; all images were generated using the same MIPs threshold. Uptake of ^111^In-mPD-L1 can be appreciated in tumor (T), spleen (S), *S. aureus*-infected (SA), lymph nodes (LN), liver (L), and brown adipose tissue B. LPS, lipopolysaccharide; Hk-*C. albicans*, heat-killed *Candida albicans*; SPECT, single-photon emission CT; MIPs, maximum intensity projections.

Increasing the antibody dose to 100 µg of ^111^In-mPD-L1 restored blood levels and tumor uptake in LPS-challenged mice to similar levels as in control mice (**Figures 1A**, **2B** and **Supplementary Table 1**). Accumulation of ^111^In-mPD-L1 in lymphoid organs remained elevated but was less pronounced as compared with the lower antibody dose. Spleen uptake in mice injected with 100 µg of ^111^In-mPD-L1 was 21.0 ± 5.0%ID/g versus 12.6 ± 1.9%ID/g (p < 0.01), for LPS-challenged versus control mice. SPECT/CT imaging confirmed the *ex vivo* biodistribution analyses for both antibody doses (**Figure 3**), namely, LPS stimulation increased ^111^In-mPD-L1 uptake in lymphoid organs and decreased tumor uptake. Furthermore, in control and LPS-stimulated mice, ^111^In-mPD-L1 accumulation in brown adipose tissue and the liver was observed.

**Figure 4:**
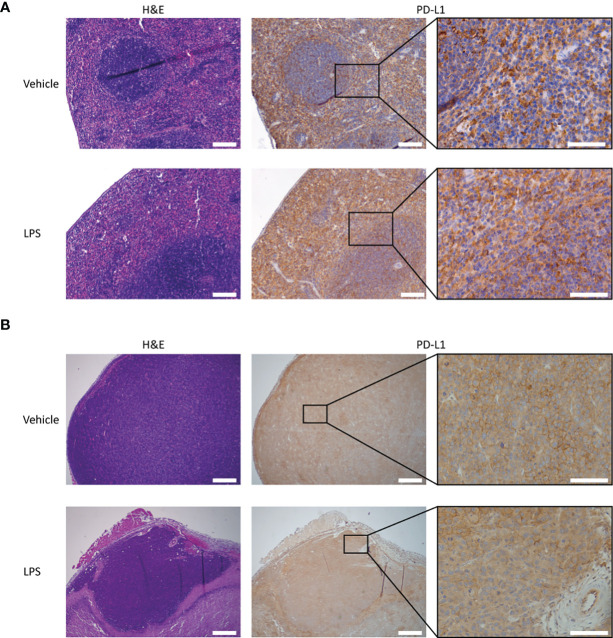
PD-L1 expression and H&E staining of tumor and splenic tissues of LPS- and vehicle-treated mice. Representative images of the immunohistochemical analysis of PD-L1 expression in tumor and spleen tissue of RenCa tumor-bearing BALB/c mice, 2 days after LPS stimulation and vehicle control injection. **(A)** Splenic PD-L1 expression is very high for both vehicle control- and LPS-treated animals. LPS stimulation appears to slightly increase PD-L1 expression in the red pulp. **(B)** Tumor PD-L1 expression is not significantly altered by immune stimulation with LPS. The scale bar represents 500 µm or 100 µm in inset. LPS, lipopolysaccharide.

To determine whether the LPS-induced changes in the blood clearance and biodistribution reflected changes in PD-L1 expression levels, and not non-specific Fc-receptor-mediated uptake and clearance, separate groups of mice were injected with an isotype control antibody (^111^In-IgG2a), using the same experimental design. In contrast to ^111^In-mPD-L1, blood clearance and *in vivo* biodistribution of both the 30 µg and 100 µg dose levels of ^111^In-IgG2a were not affected by LPS challenge (**Supplementary Table 1**). Furthermore, immunohistochemistry for PD-L1 showed that splenic PD-L1 expression was very high and that LPS stimulation might have increased its expression in the red pulp (**Figure 4**). Finally, immunohistochemical analysis of tumor sections showed that LPS stimulation did not decrease PD-L1 expression on the tumor cell membrane, indicating that the lower ^111^In-mPD-L1 tumor uptake was not due to decreased PD-L1 expression.

### ^111^In-mPD-L1 Detects Locoregional Upregulation of PD-L1 in *Staphylococcus aureus* Infection

Local intramuscular *S. aureus* infection (2.5 × 10^7^ CFU) did not affect blood clearance of ^111^In-mPD-L1 (**Figure 1**). Furthermore, ^111^In-mPD-L1 accumulation in tumor and lymphoid organs was unaffected by *S. aureus* infection (**Figure 2**). However, ^111^In-mPD-L1 uptake in *S. aureus*-infected muscle itself was significantly increased compared with vehicle-injected muscle (30 µg of ^111^In-mPD-L1: 8.6 ± 2.6%ID/g versus 1.7 ± 0.8%ID/g, respectively p < 0.001). SPECT/CT imaging confirmed these results as, apart from the accumulation of ^111^In-mPD-L1 in the *S. aureus*-infected muscle, no differences in antibody distribution were observed (**Figure 3** and **Supplementary Table 1**).

Whether the ^111^In-mPD-L1 uptake in *S. aureus* infections was PD-L1 mediated was evaluated by comparing the uptake with that of ^111^In-IgG2a. Although ^111^In-IgG2a did show uptake in *S. aureus*-infected muscles, this was twofold lower as compared with ^111^In-mPD-L1 (5.6 ± 0.6%ID/g versus 10.2 ± 1.8%ID/g, p < 0.001), indicating that uptake was predominantly PD-L1 mediated. Similarly, as for ^111^In-mPD-L1, *S. aureus* infection did not alter blood clearance, and tumor and normal tissue targeting of ^111^In-IgG2a.

**Figure 5:**
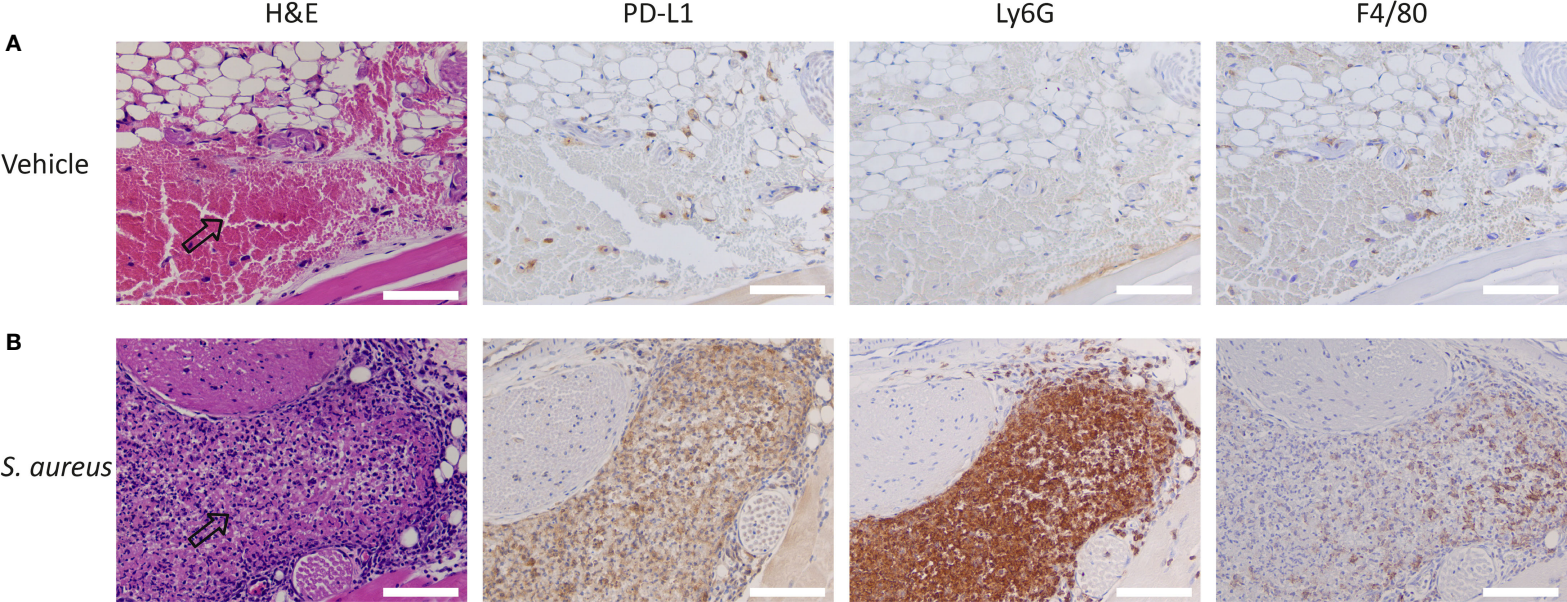
Immunohistochemistry for PD-L1, F4/80, and Ly6G expression and H&E staining of *Staphylococcus aureus*-infected and vehicle control-injected muscles. Representative images of DAB-based (brown color) immunohistochemistry on consecutive tissue sections for PD-L1, Ly6G, and F4/80 of **(A)** vehicle control-injected muscle and **(B)** *S. aureus*-infected muscle tissues of BALB/c mice. Arrows indicate sites where injected autologous blood:*S. aureus* or autologous blood:vehicle mixtures accumulated. *S. aureus* infections attract significantly higher numbers of PD-L1 positive Ly6G^+^ neutrophils and F4/80^+^ macrophages when compared with vehicle control-injected muscles. Scale bar represents 100 µm.

To further demonstrate that the accumulation observed in *S. aureus*-infected muscle was predominantly PD-L1 specific, we performed immunohistochemical analysis of the *S. aureus*-infected muscle. We Observed a high number of PD-L1 positive cells in and around sites of *S. aureus* infections, whereas only a few PD-L1 expressing cells were detected in the control site (**Figure 5**). The PD-L1 positive cells morphologically resembled neutrophils and macrophages, and staining with Ly6G and F4/80 showed that the PD-L1 expression colocalized with sites containing high numbers of neutrophils with macrophages present in fewer numbers. At the injection site of vehicle control, fewer PD-L1-positive macrophages and few to no neutrophils were detected. Both the ^111^In-IgG2a biodistribution analysis and the immunohistochemical analyses show that the uptake of ^111^In-mPD-L1 in *S. aureus*-infected muscles was partly PD-L1-mediated.

### Heat-Killed *Candida albicans* Stimulation Does Not Affect ^111^In-mPD-L1 Pharmacokinetics

The blood clearance and *in vivo* biodistribution of ^111^In-mPD-L1 in mice intraperitoneally injected with Hk-*C. albicans* was comparable to those of vehicle controls for both antibody doses (**Figures 1**, **2**). In this model, no differences in lymphoid tissue or tumor uptake of ^111^In-mPD-L1 were observed. SPECT/CT imaging confirmed these observations (**Figure 3**). Similarly, no effect on the blood clearance or biodistribution was observed for ^111^In-IgG2a.

## Discussion

PD-L1 is a central immune checkpoint molecule in tissue homeostasis and peripheral tolerance (1). Monoclonal antibodies blocking the interaction between PD-1 and PD-L1 have had a tremendous impact in the field of onco-immunology (3) and are now established treatment options for many cancer types (4). Realizing that immune checkpoint molecules are also involved in immune paralysis following severe systemic inflammatory responses (28), or persistent infections due to sustained local immune suppressions (29), PD-1 or PD-L1 targeting antibodies are currently investigated to overcome immune suppression in prolonged or severe infections (11, 12). Its dynamic and systemic expression in response to infectious stimuli cannot be determined by tissue sampling, but these aspects are relevant when developing novel treatment regimens. Furthermore, assessment of PD-L1 expression on tumor tissue samples is hampered by sampling errors and invasiveness of the procedure. Therefore, molecular imaging techniques using radiolabeled antibodies are of great interest, as they allow for longitudinal assessment of PD-L1 expression on a whole-body scale.

In this study, we demonstrate that inflammatory responses can significantly affect the pharmacokinetics of anti-PD-L1 antibodies. LPS challenge resulted in increased antibody uptake in lymphoid organs and thereby significantly accelerated blood clearance and decreased tumor targeting of ^111^In-mPD-L1. This effect was most pronounced with a low antibody dose of 30 µg and could be compensated for by increasing the antibody doses to 100 µg. This dose-dependent clearance from circulation has also been observed in clinical studies (20). Moreover, these results are in line with other studies that show that 10F.9G2 and other anti-PD-L1 antibodies exhibit target-mediated drug disposition (TMDD), meaning that these antibodies show non-linear pharmacokinetics at low doses, while increasing the dose leads to linear pharmacokinetics (30, 31). As a consequence, a saturation of tissues with high antigen levels, which are readily accessible (also described as “sink organs,” such as the spleen), is required to achieve effective target tissue uptake (31, 32). Studies with isotype control antibodies did not show LPS-induced changes in pharmacokinetics, which suggest that the changes are PD-L1-mediated and not solely caused by enhanced Fc-receptor-mediated uptake nor by other effects such as increased perfusion. Furthermore, our imaging and *ex vivo* biodistribution results are in accordance with previous flow cytometry studies that showed LPS-induced sepsis increased PD-L1 expression on monocytic and myeloid immune cells in the spleen (24, 33). Concluding, we found that LPS-induced sepsis can substantially alter anti-PD-L1 antibody pharmacokinetics, which can be monitored by PD-L1 imaging. For this purpose, lower antibody doses might be more sensitive to detect changes in PD-L1 expression levels in tissues with readily accessible PD-L1 (e.g., spleen) but might be unable to detect changes in less accessible tissues such as tumors.

In *S. aureus*-infected muscles, high uptake of the radiolabeled anti-PD-L1 antibody was detected by both *ex vivo* biodistribution and SPECT/CT imaging. This is in accordance with clinical studies observing the accumulation of ^89^Zr-atezolizumab in local inflammatory processes (14). In our study, the isotype control antibody experiment indicates that the uptake was partly non-specific, which can be explained by the enhanced permeability and retention effect caused by both *S. aureus* itself and the host’s inflammatory response against this pathogen (34). In this study, inflammatory responses to local *S. aureus* infections did not affect non-local ^111^In-mPD-L1 uptake or its blood clearance. This indicates that *S. aureus* elicited a localized inflammatory response with little to no systemically acting PD-L1-upregulating cytokines (e.g., IFN-y) or PD-L1 positive immune cells. Furthermore, we showed that the localized increase in PD-L1 expression is caused by monocytes and high numbers of neutrophils. These data are consistent with the immunological response to local *S. aureus* infections with induction of PD-L1 expression (6, 35) and rapid recruitment of monocytes, macrophages, and high numbers of neutrophils, of which the latter go into apoptosis and are phagocytosed on site after fulfilling their function (25). Importantly, our study shows that PD-L1 expression induced by the *S. aureus* elicited inflammatory response can be detected with nuclear imaging using anti-PD-L1 antibodies and that pharmacokinetics at tracer level were unaffected by local or systemic responses to local *S. aureus* infections.

Intraperitoneal Hk-*C. albicans* injections mimic candidemia, which has been shown to increase immune checkpoints, such as PD-L1, expression levels on peripheral blood mononuclear cells (5, 10). Furthermore, preclinical studies suggest that candidemia seems to respond to PD-1/PD-L1 blocking therapies, indicating involvement of these proteins (10). However, in our candidemia model using PD-L1 imaging, we did not observe additional sites of anti-PD-L1 antibody accumulation indicative of local inflammation or altered PD-L1 expression in lymphoid organs. These results seem contrary to findings of another study showing that PD-L1 is upregulated on splenic residing CD4+, NKT, and NK cells (10). However, that study used a two-hit candidemia model consisting of cecal ligation and puncture followed by intravenous injection of live *C. albicans* three days later, thereby conceivably eliciting a more severe inflammatory response than the model used in our study. Moreover, the PD-L1 upregulation in that study as measured by flow cytometry appeared to be modest.

To translate our preclinical findings to the clinical setting, factors should be considered concerning the interspecies differences in physiology, immune biology, age of onset, antibody dosing, localization and severity of infections, and the presence of comorbidities, which can all have differential effects on systemic PD-L1 expression and PD-L1 antibody distribution. Furthermore, for an optimal signal-to-noise ratio, the long circulation time of monoclonal antibodies should be taken into account, as these tracers typically require several days between tracer injection and scanning. In the current study, this interval was 1 day, whereas in clinical practice, 5–7 days is standard; however, shorter intervals of 2–3 days might be sufficient depending on the research question. As the inflammatory effects of cytokines on PD-L1 expression levels can be rapid and/or transient, a short interval between antibody injection and scanning is preferred. Therefore, the use of PD-L1 targeting tracers with shorter circulation times such as peptides or adnectins, which are already being studied clinically, can be used. Finally, PD-L1 imaging cannot differentiate between cell populations. Therefore, complementary tools such as flow cytometry, immunohistochemistry, and RNA-sequencing are necessary to answer more mechanistic questions.

This study opens the door for nuclear imaging to aid in the rational design of studies to advance immune checkpoint inhibition to overcome immune dysfunction in patients with chronic infectious diseases and severe septic responses. For example, clinical imaging can facilitate in determining target tissue antibody uptake in pharmacological studies, thereby supporting and streamlining optimal dose finding and development of treatment regimens. Second, it can be used to evaluate possible effects on PD-L1 expression levels and consequently the potential for therapeutic synergy of PD-L1 blockade and current antimicrobial immune-activating therapies such as prednisone, TNF-alpha antagonists, and IL-6 blockers.

In summary, we demonstrate that local and systemic infectious triggers can alter anti-PD-L1 antibody pharmacokinetics using ^111^In-mPD-L1 SPECT/CT imaging. Increasing the anti-mPD-L1 antibody dose can normalize tumor targeting in mice with septic inflammatory responses. In the future, PD-L1 imaging can be used as a suitable tool to explore host responses in infectious conditions.

## Data Availability Statement

The raw data supporting the conclusions of this article will be made available by the authors, without undue reservation.

## Ethics Statement

The animal study was reviewed and approved by the Animal Welfare Body of the Radboud University, Nijmegen, and the Dutch Central Authority for Scientific Procedures on Animals.

## Author Contributions

GS, SH, GA, JB, and EA contributed to the conception and design of the study. GS and JM performed the experiments. GS organized the database. GS performed the statistical analysis. GS wrote the first draft of the manuscript. SH and EA wrote sections of the manuscript. All authors contributed to manuscript revision and read and approved the submitted version.

## Funding

This project received funding from the Netherlands Organization for Scientific Research (NWO, project number 91617039) and the Dutch Cancer Society (KWF, project number 10099).

## Conflict of Interest

The authors declare that the research was conducted in the absence of any commercial or financial relationships that could be construed as a potential conflict of interest.

## Publisher’s Note

All claims expressed in this article are solely those of the authors and do not necessarily represent those of their affiliated organizations, or those of the publisher, the editors and the reviewers. Any product that may be evaluated in this article, or claim that may be made by its manufacturer, is not guaranteed or endorsed by the publisher.

## Abbreviations

PD-L1, programmed death-ligand 1; PD-1, programmed cell death protein 1; LPS, lipopolysaccharide; Hk-*C. albicans*, heat-killed *Candida albicans*; *S. aureus*, *Staphylococcus aureus*; IFN-γ, interferon gamma; TNF-α, tumor necrosis factor alpha; ^111^In-mPD-L1, indium-111-labeled anti-mouse PD-L1 antibody; ^111^In-rIgG2a, indium-111-labeled rat IgG2a antibody; Ict-DTPA, isothiocyanatobenzyl-diethylenetriaminepentaacetic acid; MBq, megabequerel; A_s_, specific activity; EDTA, ethylenediaminetetraacetic; SPECT, single-photon emission CT; iTLC, instant thin-layer chromatography; CFU, colony-forming units; i.v., intravenous; i.p., intraperitoneal; i.m., Intramuscular; Pi, post injection; RT, room temperature; TMDD, target-mediated drug disposition; AR, antigen retrieval; PBS, phosphate-buffered saline; BSA, bovine serum albumin; NrabS, normal rabbit serum; (m)Ab, (monoclonal) antibody; v/v, volume per volume.
