# Supplementary material for: PD-L1 Antibody Pharmacokinetics and Tumor Targeting in Mouse Models for Infectious Diseases
Source: Front Immunol. 2022 Mar 10;13:837370. doi: 10.3389/fimmu.2022.837370 (PMC8960984; doi:10.3389/fimmu.2022.837370)
Supplement: Supplementary file 1 [file DataSheet_1.docx]

Supplementary Material

**Supplemental table 1.** *Ex vivo* biodistribution results of 30 and 100 µg ^111^In-mPD-L1 and ^111^In-IgG2a in LPS, Hk-*C. albicans*, *S. aureus* infected and vehicle injected RenCa tumor-bearing mice. Mean results and standard deviation per tissue are shown in % injected dose per gram (%ID/g, mean ± sd).

| 30 µg ^111^In-mPD-L1 | Vehicle  (n= 6) | LPS  (n= 5) | Hk-  *C. albicans*  (n= 6) | *S. aureus* vehicle  (n= 4) | *S. aureus*  (n= 4) |
| --- | --- | --- | --- | --- | --- |
|  | (%ID/g) | (%ID/g) | (%ID/g) | (%ID/g) | (%ID/g) |
| Blood 1h | 35.5 ± 6.8 | 31.4 ± 6.8 | 37.1 ± 10.1 | 37.3 ± 3.3 | 33.5 ± 4.8 |
| Blood 4h | 27.4 ± 5.0 | 21.5 ± 4.0 | 26.1 ± 6.7 | 27.2 ± 3.4 | 25.0 ± 4.8 |
| Blood 24h | 11.5 ± 7.2 | 3.6 ± 1.6 | 7.8 ± 3.0 | 10.7 ± 3.6 | 8.7 ± 2.7 |
| Tumor | 25.2 ± 5.2 | 8.1 ± 4.5 | 21.9 ± 4.6 | 22.3 ± 5.9 | 16.7 ± 2.4 |
| Spleen | 21.2 ± 6.9 | 49.9 ± 4.4 | 21.0 ± 4.2 | 23.8 ± 4.2 | 22.5 ± 4.8 |
| Brown adipose tissue | 18.5 ± 3.8 | 23.8 ± 3.3 | 17.0 ± 2.2 | 19.8 ± 6.8 | 16.3 ± 3.0 |
| Lymph nodes | 17.3 ± 6.1 | 33.8 ± 9.6 | 24.4 ± 13.5 | 18.3 ± 14.1 | 21.1 ± 4.3 |
| Duodenum | 11.0 ± 3.4 | 19.3 ± 5.9 | 14.3 ± 4.0 | 15.9 ± 3.3 | 9.8 ± 4.4 |
| Thymus | 9.0 ± 3.9 | 8.8 ± 4.6 | 6.9 ± 1.3 | 7.6 ± 3.3 | 7.7 ± 3.0 |
| Bone marrow | 7.4 ± 2.3 | 17.8 ± 3.3 | 5.3 ± 1.1 | 7.6 ± 1.3 | 9.6 ± 4.1 |
| Bone | 1.6 ± 0.3 | 3.8 ± 0.7 | 1.8 ± 0.3 | 1.8 ± 0.6 | 1.6 ± 0.4 |
| Heart | 5.5 ± 1.4 | 8.8 ± 1.8 | 5.1 ± 0.3 | 4.6 ± 0.3 | 4.1 ± 0.4 |
| Lung | 12.1 ± 2.4 | 17.5 ± 1.9 | 11.0 ± 1.8 | 11.1 ± 3.3 | 10.4 ± 2.1 |
| Pancreas | 2.6 ± 0.3 | 4.0 ± 1.0 | 4.6 ± 2.3 | 2.2 ± 0.4 | 2.4 ± 0.9 |
| Stomach | 3.3 ± 0.7 | 4.7 ± 0.9 | 3.8 ± 0.6 | 3.0 ± 0.4 | 2.8 ± 0.7 |
| Colon | 4.7 ± 1.0 | 4.2 ± 0.7 | 5.0 ± 0.7 | 4.3 ± 0.5 | 6.1 ± 3.8 |
| Liver | 13.8 ± 4.6 | 13.2 ± 2.2 | 11.4 ± 1.7 | 12.8 ± 2.5 | 10.1 ± 1.9 |
| Kidney | 9.3 ± 1.9 | 13.9 ± 2.9 | 8.4 ± 1.0 | 8.6 ± 0.6 | 7.5 ± 1.2 |
| Muscle | 1.5 ± 0.3 | 1.8 ± 0.3 | 1.6 ± 1.1 | 1.3 ± 0.1 | 1.5 ± 0.6 |
| *S. aureus* infection | n/a | n/a | n/a | 1.7 ± 0.8 | 8.6 ± 2.6 |
|  |  |  |  |  |  |
| 100 µg ^111^In-mPD-L1 | **Vehicle**  **(n= 6)** | **LPS**  **(n= 5)** | **Hk-C. albicans**  **(n= 5)** | ***S. aureus* vehicle**  **(n= 5)** | ***S. aureus***  **(n= 4)** |
|  | (%ID/g) | (%ID/g) | (%ID/g) | (%ID/g) | (%ID/g) |
| Blood 1h | 36.8 ± 7.0 | 36.8 ± 4.4 | 29.3 ± 1.1 | 35.9 ± 8.2 | 32.0 ± 4.0 |
| Blood 4h | 30.4 ± 6.0 | 28.6 ± 3.1 | 26.2 ± 2.5 | 29.3 ± 3.8 | 25.3 ± 3.0 |
| Blood 24h | 11.7 ± 4.0 | 10.7 ± 1.7 | 11.3 ± 3.4 | 13.0 ± 3.3 | 12.3 ± 0.9 |
| Tumor | 21.4 ± 5.6 | 17.3 ± 9.1 | 20.4 ± 9.2 | 19.5 ± 3.9 | 17.4 ± 2.5 |
| Spleen | 12.6 ± 1.9 | 21.0 ± 5.0 | 10.7 ± 3.0 | 12.3 ± 1.4 | 12.0 ± 1.6 |
| Brown adipose tissue | 10.2 ± 2.3 | 12.5 ± 1.0 | 9.6 ± 1.7 | 10.1 ± 1.0 | 9.7 ± 0.9 |
| Lymph nodes | 8.8 ± 4.6 | 20.3 ± 3.5 | 11.3 ± 4.8 | 12.3 ± 3.9 | 12.7 ± 3.1 |
| Duodenum | 6.4 ± 2.1 | 11.3 ± 0.8 | 5.9 ± 1.2 | 6.9 ± 1.2 | 7.0 ± 2.4 |
| Thymus | 6.3 ± 1.2 | 8.6 ± 1.5 | 6.2 ± 0.7 | 6.6 ± 0.4 | 6.9 ± 0.8 |
| Bone marrow | 5.9 ± 1.6 | 11.1 ± 1.4 | 5.3 ± 0.8 | 5.8 ± 0.7 | 5.6 ± 1.2 |
| Bone | 1.7 ± 0.3 | 2.4 ± 0.4 | 1.4 ± 0.3 | 1.5 ± 0.3 | 1.6 ± 0.3 |
| Heart | 4.3 ± 0.6 | 5.2 ± 0.3 | 3.8 ± 0.5 | 4.2 ± 0.4 | 3.8 ± 0.6 |
| Lung | 12.8 ± 2.2 | 12.9 ± 1.6 | 11.2 ± 1.8 | 11.4 ± 1.4 | 11.0 ± 2.0 |
| Pancreas | 1.9 ± 0.2 | 3.3 ± 0.5 | 3.1 ± 0.7 | 2.1 ± 0.4 | 2.1 ± 0.3 |
| Stomach | 2.3 ± 0.2 | 3.4 ± 0.4 | 2.4 ± 0.4 | 2.0 ± 0.4 | 2.1 ± 0.5 |
| Colon | 3.0 ± 0.8 | 3.6 ± 0.9 | 3.0 ± 0.7 | 2.7 ± 0.2 | 2.5 ± 0.3 |
| Liver | 13.4 ± 5.9 | 9.3 ± 1.2 | 10.6 ± 1.8 | 12.3 ± 2.2 | 9.9 ± 1.1 |
| Kidney | 7.4 ± 0.8 | 8.4 ± 0.7 | 7.2 ± 0.7 | 7.6 ± 0.5 | 6.6 ± 0.8 |
| Muscle | 1.0 ± 0.3 | 1.4 ± 0.2 | 1.0 ± 0.2 | 1.2 ± 0.2 | 1.2 ± 0.3 |
| *S. aureus* infection | n/a | n/a | n/a | 1.2 ± 0.2 | 10.2 ± 1.8 |

| 30 µg ^111^In-IgG2a | Vehicle  (n= 6) | LPS  (n= 5) | Hk-  *C. albicans*  (n= 6) | *S. aureus* vehicle  (n= 4) | *S. aureus*  (n= 4) |
| --- | --- | --- | --- | --- | --- |
|  | (%ID/g) | (%ID/g) | (%ID/g) | (%ID/g) | (%ID/g) |
| Blood 1h | 36.7 ± 3.8 | 39.3 ± 1.0 | 40.9 ± 3.8 | 36.8 ± 4.4 | 43.2 ± 6.6 |
| Blood 4h | 24.4 ± 1.8 | 26.7 ± 0.5 | 26.2 ± 2.4 | 25.0 ± 2.8 | 27.6 ± 4.4 |
| Blood 24h | 14.2 ± 2.0 | 12.4 ± 0.8 | 15.2 ± 2.0 | 12.3 ± 1.2 | 13.7 ± 2.0 |
| Tumor | 6.3 ± 1.1 | 6.5 ± 1.2 | 8.2 ± 1.5 | 6.3 ± 1.0 | 6.5 ± 1.5 |
| Spleen | 2.5 ± 0.2 | 3.7 ± 0.2 | 3.0 ± 0.5 | 2.4 ± 0.5 | 2.7 ± 0.3 |
| Brown adipose tissue | 2.9 ± 0.6 | 3.3 ± 0.5 | 3.8 ± 1.0 | 2.4 ± 0.8 | 3.4 ± 0.5 |
| Lymph nodes | 2.9 ± 0.3 | 3.9 ± 0.6 | 3.6 ± 1.0 | 2.5 ± 0.4 | 2.9 ± 0.8 |
| Duodenum | 2.6 ± 1.0 | 3.0 ± 0.6 | 2.5 ± 0.5 | 2.1 ± 0.3 | 2.2 ± 0.3 |
| Thymus | 3.3 ± 0.7 | 4.3 ± 1.2 | 3.9 ± 1.4 | 2.9 ± 0.4 | 4.1 ± 0.4 |
| Bone marrow | 3.1 ± 0.6 | 3.4 ± 0.7 | 3.6 ± 0.6 | 2.9 ± 0.3 | 3.1 ± 0.4 |
| Bone | 0.9 ± 0.2 | 1.3 ± 0.1 | 1.0 ± 0.2 | 1.0 ± 0.1 | 1.1 ± 0.1 |
| Heart | 3.7 ± 0.3 | 4.3 ± 0.6 | 4.4 ± 0.8 | 3.3 ± 0.4 | 4.1 ± 0.9 |
| Lung | 12.1 ± 1.6 | 9.7 ± 1.6 | 13.1 ± 1.6 | 12.0 ± 1.4 | 9.0 ± 1.1 |
| Pancreas | 1.6 ± 0.2 | 2.8 ± 0.1 | 2.5 ± 0.5 | 1.5 ± 0.3 | 1.8 ± 0.1 |
| Stomach | 1.8 ± 0.2 | 2.4 ± 0.3 | 2.2 ± 0.5 | 2.0 ± 0.3 | 2.1 ± 0.2 |
| Colon | 2.0 ± 0.4 | 2.5 ± 0.2 | 2.3 ± 0.5 | 1.9 ± 0.4 | 2.7 ± 1.3 |
| Liver | 3.9 ± 0.7 | 4.1 ± 0.3 | 3.9 ± 0.6 | 3.5 ± 1.1 | 4.1 ± 0.9 |
| Kidney | 6.3 ± 0.5 | 6.8 ± 0.5 | 7.1 ± 0.9 | 6.2 ± 0.9 | 7.1 ± 0.4 |
| Muscle | 0.7 ± 0.1 | 1.0 ± 0.1 | 0.9 ± 0.2 | 1.0 ± 0.4 | 1.0 ± 0.2 |
| *S. aureus* infection | n/a | n/a | n/a | 1.1 ± 0.4 | 5.3 ± 0.9 |
|  |  |  |  |  |  |
| 100 µg ^111^In-IgG2a | **Vehicle**  **(n= 4)** | **LPS**  **(n= 4)** | **Hk-**  ***C. albicans***  **(n= 6)** | ***S. aureus* vehicle**  **(n= 6)** | ***S. aureus***  **(n= 5)** |
|  | (%ID/g) | (%ID/g) | (%ID/g) | (%ID/g) | (%ID/g) |
| Blood 1h | 46.2 ± 8.4 | 45.3 ± 9.2 | 38.2 ± 3.9 | 38.3 ± 5.1 | 38.6 ± 2.5 |
| Blood 4h | 30.4 ± 6.3 | 30.4 ± 4.9 | 25.2 ± 1.1 | 23.8 ± 2.9 | 26.2 ± 2.7 |
| Blood 24h | 17.2 ± 2.3 | 14.3 ± 1.7 | 15.5 ± 1.7 | 13.8 ± 1.2 | 12.9 ± 1.3 |
| Tumor | 7.2 ± 0.7 | 9.0 ± 1.6 | 8.1 ± 2.8 | 7.0 ± 1.3 | 5.4 ± 0.7 |
| Spleen | 2.7 ± 0.3 | 2.8 ± 0.7 | 3.0 ± 0.3 | 2.8 ± 0.4 | 2.9 ± 0.5 |
| Brown adipose tissue | 2.6 ± 0.3 | 3.6 ± 0.9 | 3.2 ± 0.6 | 3.1 ± 0.9 | 3.2 ± 0.7 |
| Lymph nodes | 3.2 ± 0.6 | 3.0 ± 0.3 | 3.4 ± 0.5 | 3.0 ± 0.5 | 3.7 ± 0.9 |
| Duodenum | 2.9 ± 0.5 | 2.7 ± 0.7 | 2.8 ± 0.7 | 2.4 ± 0.6 | 2.7 ± 0.5 |
| Thymus | 3.0 ± 0.4 | 4.2 ± 1.1 | 3.5 ± 0.3 | 3.0 ± 0.6 | 3.1 ± 0.6 |
| Bone marrow | 3.0 ± 0.6 | 3.5 ± 0.7 | 3.5 ± 0.8 | 3.7 ± 0.6 | 2.9 ± 0.2 |
| Bone | 0.9 ± 0.1 | 1.0 ± 0.2 | 1.1 ± 0.1 | 1.0 ± 0.2 | 1.1 ± 0.1 |
| Heart | 4.0 ± 0.4 | 5.2 ± 1.4 | 4.3 ± 0.6 | 4.1 ± 0.7 | 3.9 ± 0.6 |
| Lung | 12.9 ± 1.7 | 9.0 ± 0.7 | 13.6 ± 1.7 | 13.3 ± 1.5 | 10 ± 1.1 |
| Pancreas | 1.9 ± 0.3 | 2.2 ± 0.1 | 2.3 ± 0.3 | 1.6 ± 0.3 | 2.1 ± 0.6 |
| Stomach | 2.1 ± 0.3 | 2.3 ± 0.4 | 2.3 ± 0.3 | 1.9 ± 0.3 | 2.6 ± 1.0 |
| Colon | 2.0 ± 0.5 | 2.6 ± 0.8 | 2.7 ± 0.1 | 2.1 ± 0.7 | 2.3 ± 0.6 |
| Liver | 3.2 ± 0.4 | 3.7 ± 0.1 | 3.6 ± 0.5 | 3.7 ± 0.6 | 3.8 ± 0.7 |
| Kidney | 7.3 ± 0.5 | 7.0 ± 0.4 | 7.1 ± 0.4 | 6.7 ± 0.8 | 7.2 ± 0.5 |
| Muscle | 0.8 ± 0.1 | 1.0 ± 0.2 | 0.8 ± 0.1 | 1.0 ± 0.1 | 0.9 ± 0.1 |
| *S. aureus* infection | n/a | n/a | n/a | 0.9 ± 0.1 | 5.6 ± 0.6 |
